# Effects of an Eight-Week Superimposed Submaximal Dynamic Whole-Body Electromyostimulation Training on Strength and Power Parameters of the Leg Muscles: A Randomized Controlled Intervention Study

**DOI:** 10.3389/fphys.2018.01719

**Published:** 2018-12-05

**Authors:** Florian Micke, Heinz Kleinöder, Ulrike Dörmann, Nicolas Wirtz, Lars Donath

**Affiliations:** Department of Intervention Research in Exercise Training, German Sport University Cologne, Cologne, Germany

**Keywords:** whole-body EMS, electrical stimulation, strength training, MVC, peak power output

## Abstract

The purpose of this study was to assess the effects of dynamic superimposed submaximal whole-body electromyostimulation (WB-EMS) training on maximal strength and power parameters of the leg muscles compared with a similar dynamic training without WB-EMS. Eighteen male sport students were randomly assigned either to a WB-EMS intervention (INT; *n* = 9; age: 28.8 (*SD*: 3.0) years; body mass: 80.2 (6.6) kg; strength training experience: 4.6 (2.8) years) or a traditional strength training group (CON; *n* = 9; age: 22.8 (2.5) years; body mass: 77.6 (9.0) kg; strength training experience: 4.5 (2.9) years). Both training intervention programs were performed twice a week over a period of 8 weeks with the only difference that INT performed all dynamic exercises (e.g., split squats, glute-ham raises, jumps, and tappings) with superimposed WB-EMS. WB-EMS intensity was adjusted to 70% of the individual maximal tolerable pain to ensure dynamic movement. Before (PRE), after (POST) and 2 weeks after the intervention (FU), performance indices were assessed by maximal strength (F_max_) and maximal power (P_max_) testing on the leg extension (LE), leg curl (LC), and leg press (LP) machine as primary endpoints. Additionally, vertical and horizontal jumps and 30 m sprint tests were conducted as secondary endpoints at PRE, POST and FU testing. Significant time effects were observed for strength and power parameters on LE and LC (LE F_max_ +5.0%; LC P_max_ +13.5%). A significant time × group interaction effect was merely observed for F_max_ on the LE where follow-up *post hoc* testing showed significantly higher improvements in the INT group from PRE to POST and PRE to FU (INT: +7.7%, *p* < 0.01; CON: +2.1%). These findings indicate that the combination of dynamic exercises and superimposed submaximal WB-EMS seems to be effective in order to improve leg strength and power. However, in young healthy adults the effects of superimposed WB-EMS were similar to the effects of dynamic resistance training without EMS, with the only exception of a significantly greater increase in leg extension F_max_ in the WB-EMS group.

## Introduction

Health-related strength training recommendations regarding intensity, frequency and volume of strength training for maximal strength gains in trained individuals refer to 80–85% of One-repetition maximum, 2 days per week with a volume of 3–8 sets per muscle group (Peterson et al., 2005). If strength training is incorporated into fitness programs, it can also improve cardiovascular functions and psychological well-being, prevent osteoporosis and promote both weight loss and maintenance (Ratamess et al., 2009). Appropriate maximal strength and power training is also considered crucial for sport-specific physical development in terms of speed, dynamics and injury prevention (Reilly, 2007; Sander et al., 2013).

Electromyostimulation (EMS), an training technology for intensifying the training load, is known to be an effective and appealing complementary add-on training method to potentially further improve athletic performance factors (Filipovic et al., 2012). The benefits of EMS training can be attributed to the accentuated activation of fast motor units at relatively low force levels due to a non-selective recruitment pattern (Gregory and Bickel, 2005). Furthermore, EMS potentially supports the athlete in achieving greater strength and power adaptations by a synchronous recruitment of muscle fibers and an increased firing rate (Gregory and Bickel, 2005).

Most of the previous studies in the field of EMS examined isometric contractions of local muscles with maximal stimulation intensities at the individual pain threshold (Filipovic et al., 2011). Intervention studies using isometric EMS revealed considerable gains in isometric strength of about +32% in trained athletes (Filipovic et al., 2012). However, not only the increase in strength and power is essential. The functional transfer of these gains into sport-specific movements, especially in competitive sports is even more relevant. A sport-specific orientation of EMS training with dynamic exercises could account for the requirements of athletes, in order to achieve this functional transfer (Cormie et al., 2011a,b). Isometric EMS with maximal stimulation intensities does not meet the movement specificity required for the completion of sports movements (Paillard et al., 2005). When superimposed EMS is applied onto voluntary contractions, force was higher with respect to voluntary actions at eccentric actions (Paillard et al., 2005). In concentric and isometric actions, voluntary activation evoked higher force than EMS (Paillard et al., 2005). However, most recent evidence suggests that EMS superimposed onto voluntary contractions in a submaximal task could result in greater muscle fibers recruitment compared with voluntary or electrical stimulation alone and would be likely to generate greater gains of motor output after a training period (Paillard, 2018). Moreover, a low voluntary movement control exists at maximum stimulation intensities (Babault et al., 2007) and only submaximal contractions enable an efficient movement control with superimposed EMS (Bezerra et al., 2011). As a consequence, only stimulation intensities below the individual pain threshold allow dynamic movements with superimposed EMS. Taking the transfer of strength and power to sport-specific movements into account, it is well documented that a combination of separate EMS training and separate dynamic sport-specific exercises like jumping and sprinting could lead to these transfer effects (Maffiuletti et al., 2002; Herrero et al., 2006).

Compared to a local EMS, Whole-Body-Electromyostimulation (WB-EMS) stimulates several muscle groups like muscle-chains or agonist/antagonist simultaneously during dynamic movements. In non-athletic adults it is known that WB-EMS improves muscle mass and function while reducing fat mass and low back pain (Kemmler et al., 2018). At least in athletes, there is some evidence that locally applied EMS was slightly more favorable for increasing strength-related outcomes compared with WB-EMS (Kemmler et al., 2018). However, stimulation of muscle chains could support dynamic movements by compensating usual weak points like hip extensor (Lynn and Noffal, 2012) or lower back muscles (Hamlyn et al., 2007). It is assumed that a simultaneously and counterproductive activation of agonist and antagonist evokes additional demands on voluntary contraction, especially on a reduced co-activation of antagonistic muscles to continue dynamic exercises with superimposed EMS (Wirtz et al., 2016).

Due to the aforementioned background, the question arises whether a simultaneous combination of submaximal WB-EMS and dynamic strength and/or sport-specific exercises leads to improvements in both strength and power as well as jumping and sprinting performance. On the one hand, an advantage of this training approach is indicated by strength training over the entire muscle length. On the other hand it might induce beneficial effects due to the intensification of the technique training. Therefore, the aim of this study was to evaluate the effects of an 8-week, 16-session training program using dynamic submaximal WB-EMS training compared with traditional voluntary dynamic strength training without WB-EMS on (1) maximal strength and maximal power parameters and (2) on jumping and sprinting performance in male adult sport students. We hypothesized that the use of dynamic submaximal superimposed WB-EMS provides greater training adaptations and improves performance to a greater extent compared to traditional dynamic strength training without WB-EMS alone.

## Materials and Methods

### Study Design

**FIGURE 1:**
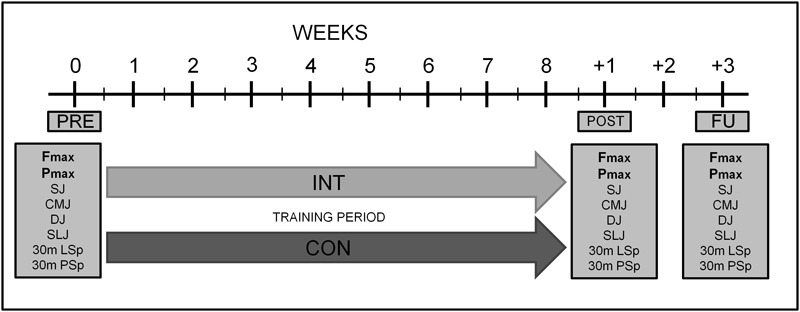
Schematic representation of the experimental protocol. F_max_, maximal isometric force; P_max_, maximal isoinertial power; SJ, Squat Jump; CMJ, Counter Movement Jump; DJ, Drop Jump; SLJ, Standing Long Jump; 30 m LSp, 30 m Linear Sprint; 30 m PSp, 30 m Pendulum Sprint; INT, WB-EMS Intervention Group; CON, Traditional strength training Group.

This study was designed as a 2-armed parallel-group, randomized controlled trial comparing the effects of submaximal superimposed dynamic WB-EMS (INT) with the effects of dynamic strength training without WB-EMS (CON) (Figure 1). The INT and CON groups completed 16 training sessions in 8 weeks twice a week. To determine training effects, isometric strength and isoinertial power diagnostics as well as jumping and sprinting tests were conducted under constant and stable lab conditions. Measurements of the primary and secondary outcome took place before the training period (PRE), after the training period (POST) and 2 weeks after the training period as follow-up (FU). PRE-, POST-, and FU-testings were intra-individually performed at the similar time of the day. After PRE, the subjects were randomly assigned (minimization method, strata: age, strength training experience) to either INT or CON. In order to minimize influences of unspecific training loads, both groups were asked to refrain from any changes of their habitual physical activity behavior. Furthermore, all participants were instructed to maintain their normal dietary intake before and during the study.

### Participants

Twenty male sport students volunteered to participate in the study. Inclusion criteria were: young adults between 18 and 30 years, who had a medical certificate attesting full physical fitness, had at least 2 years of strength training experience and had a sporting background in sports which requires performance abilities such as sprinting and/or jumping (e.g., soccer, handball, basketball, football, track and field, tennis). Exclusion criteria were any training experiences in WB-EMS. One week before the PRE tests, a familiarization session for testing was conducted. Thereby, the testing devices were adjusted and the participants were familiarized with the testing procedures. After the randomization, participants of the INT group were familiarized with WB-EMS and the training intensity was determined. Written informed consent was obtained from all participants after giving comprehensive study instructions. The study protocol was approved by the “Ethics Committee of the German Sport University Cologne” and complied with the Declaration of Helsinki. Two participants, one in each group, had to terminate study participation due to injuries that were not related to the study. Finally, 18 participants completed all tests and the attendance rate for the training sessions was 100% for both groups (participants characteristics are presented in Table 1).

**Table 1:** Demographic variables mean (SD).

	N	Age (years)	Height (cm)	Weight (kg)	BMI (kg/m^2^)	Strength training exp. (years)
**INT**	9	22.8 (3.0)	179.4 (5.1)	80.2 (6.6)	22.3 (1.8)	4.6 (2.8)
**CON**	9	22.8 (2.5)	184.9 (9.1)	77.6 (9.1)	20.9 (1.5)	4.5 (2.9)

### Training Procedure

During the 8-week training period, participants of both groups similarly performed 16 training sessions (TS) twice a week with the only difference that the INT group performed all exercises with additional superimposed WB-EMS. Each session consisted of 5 training exercises in total, 2 strength exercises for either leg extension or knee flexion (e.g., split squats, glute-ham raises) plus 3 dynamic (a) jumping exercises (e.g., hurdle jumps, lateral jumps; in the first TS of the week) or (b) sprinting exercises (e.g., resistance band running, ABC-running drills; in the second TS of the week). Training variables like intensity, number of repetitions, repetition velocity or exercises changed after the first 3 weeks and after the second 3 weeks, due to the progression principles of strength training (see Supplementary Tables S1–S6). Three sets of each training exercise were conducted. The number of repetitions differed between the exercises and ranged from 5 to 10 repetitions. Movement velocity and range of motion (ROM) were predetermined for every single training exercise and were controlled with a metronome and with markings, respectively. The training intensity of each set was recorded using the Borg Rating of Perceived Exertion (RPE) and set to >16 (> “hard”) (Tiggemann et al., 2010). A rest interval of at least 48 h between each training session was complied.

### WB-EMS

The INT group performed all exercises with additional superimposed WB-EMS. Surface electrodes (miha bodytec, Augsburg, Germany) were applied to the leg and trunk muscles. An electrode vest with fixed surface electrodes provided the stimulation of the upper body including the chest (electrode size: 15 cm length × 4.5 cm width), the upper back (23 × 10 cm), the lower back (14 × 11 cm), the latissimus (14 × 9 cm) and the abdominals (23 × 10 cm). A belt system provided the stimulation for the lower body including the muscles of the glutes (13 × 10 cm), the thighs (44 × 4 cm), and the calves (27 × 4 cm). The sizes of the electrode vest and the belt electrodes (small/medium/large) were selected according to the body size of each participant.

The WB-EMS intervention was complied with the guidelines for a safe and effective WB-EMS training (Kemmler et al., 2016a). The intensity of EMS during the training was adjusted to 70% of the individual maximal tolerable pain [maximum tolerated amperage (0–120 mA)] as previously described in detail elsewhere (Wirtz et al., 2016). The maximal tolerated amperage was determined separately for each pair of electrodes before every training session. Firstly, maximum intensity was verified for simultaneous stimulation of all muscle groups. Then, the intensity was subsequently downregulated with the main controller at the WB-EMS device to an intensity of 70% to enable dynamic movements. Impulse frequency was set at 85 Hz, pulse duration at 350 μs, impulse type was bipolar and rectangle (Kemmler et al., 2014; Filipovic et al., 2016; Kemmler et al., 2016b; Wirtz et al., 2016). On/off-time was individually adjusted within each exercise (see Supplementary Tables S1–S6). In general, EMS was applied during all the execution time of each exercise and stopped during the rest period.

### Testing Procedures

#### Strength and Power Testing

Maximal isometric strength (F_max_) and maximal isoinertial power (P_max_) diagnostics for the leg muscles were conducted on the leg extension (LE), leg curl (LC), and leg press (LP) machines (Edition-Line, gym80, Gelsenkirchen, Germany). All machines were equipped with the digital measurement technique Digimax (mechaTronic, Hamm, Germany). The multi-channel measuring system consisted of a force and distance sensor (megaTron, Munich, Germany), a PC-2-Channel-Interface, a computer with serial port and measurement/analysis software (IsoTest 2.0 and DynamicTest 2.0). F_max_ [N] and P_max_ [W] were calculated for statistical analysis and data presentation. Each participant performed 3 isometric and 3 isoinertial test attempts on the each leg machine. For F_max_ and P_max_, the attempt with the highest value was subsequently used for further analysis. Isometric tests were conducted at an inner knee angle of 120 degree on the LE as well as LP and 150 degrees on the LC. Isoinertial test attempts were conducted with an additional load. This load was individually calculated as a percentage of the F_max_ in a further isometric test with the same angle as the starting position of the isoinertial test (LE and LP 90°; LC 170°). The attempts were conducted with 40% additional load on the LC and with 60% additional load on the LP as well as on the LE over a concentric ROM (inner knee ROM: LE and LP 90–180°; LP 170–80°). The instruction was to press against the lever arm “as hard and fast as possible” (Maffiuletti et al., 2016). Strength and power parameters were considered as primary endpoints.

#### Jumping Tests

Jumping performance was quantified using the Optojump system (Microgate, Bolzano, Italy). Therefore, jump height was assessed using the flight time method. After one familiarization jump trial, the participants performed 3 trials of each jump variation [1. squat jump (SJ), 2. counter movement jump (CMJ), 3. drop jump (DJ), and 4. Standing long jump (SLJ)] in a fixed order. For the respective jump task, the highest or longest jump was used for subsequent analysis. For the SJ, participants were instructed to start jumping from a static squatted position holding the knees at 90 degrees without any preliminary movement. For the CMJ, participants were instructed to start the jump from an upright standing position, squatting down to a knee angle of approximately 90 degrees in order to jump explosively as high as possible. DJs were performed from a 38 cm box (Bobbert et al., 1987). Participants were instructed to step down from the box and then to try to jump as high as possible after a short contact time on the ground. Hands remained akimbo for the entire movement of each vertical jump in order to eliminate any influence of the arm swing. The DJ height (DJH) and the DJ contact time (DJCT) were measured. For the SLJ, the participants started the horizontal jump from an upright standing position. They were instructed to gain adequate momentum by squatting down in order to jump as far as possible and to complete the jump with a controlled landing. Jump length was determined by measuring from tip to the participants’ rear-most heel.

#### Sprinting Tests

Sprinting tests were conducted in an indoor hockey hall with a non-slippery floor. Performance was tested with a linear 30 m sprint (30 mLSp) and a pendulum sprint of 3 × 10 m (30mPSp) with 2 changes of direction of 180° (at 10 and 20 m) (Filipovic et al., 2016; Wirtz et al., 2016). Final sprint time was measured at 30 m. Starting position was 50 cm in front of the starting light beam for both sprint tests. Participants had 2 min recovery between the trials. Double infrared photoelectric barriers with a radio transmitter (DLS/F03, Sportronic, Leutenbach-Nellmersbach, Germany) were used for time measurement. The fastest time of 3 trials per sprint variation was used for subsequent analysis. Jumping and sprinting tests were conducted as secondary endpoints.

### Statistical Analysis

Data were given as means with standard deviations (SD). Statistical analyses were performed by using a statistics software package (IBM SPSS Statistics, Version 25.0, Armonk, NY, United States). All parameters were normally distributed (Shapiro–Wilk test) and variances were homogeneous (Levene test). Then, separate 2 (group: INT, CON) × 3 (time: PRE, POST, and FU) repeated measures analyses of variances (rANOVA) were calculated. Thereby, PRE values of the respective primary or secondary outcome was included as covariate in order to adjust for possible baseline differences (Vickers and Altman, 2001). In case of a significant time × group interaction, Bonferroni *post hoc* tests and standardized mean differences (SMD) were calculated for pairwise comparison. The magnitude of SMD was classified according to the following scale: 0–0.19, negligible effect; 0.20–0.49, small effect; 0.50–0.79, moderate effect; and ≥0.80, large effect (Cohen, 1988). To estimate overall time and interaction effect sizes, partial eta squared ($\eta_{p}^{2}$) was computed with $\eta_{p}^{2}$ ≥ 0.01 indicating small, ≥0.059 medium and ≥0.138 large effects (Cohen, 1988). The level of significance was set at *p* < 0.05.

## Results

### Strength and Power Parameter

F_max_ and P_max_ values for both groups are provided in Table 2. All data are adjusted for baseline differences. A significant and large time × group interaction was merely observed for F_max_ on the LE (*p* = 0.029; $\eta_{p}^{2}$ = 0.21) where *post hoc* comparisons indicated higher improvements in the INT group from PRE to POST (INT: +6.9%, *p* < 0.01; CON: +0.5%) and PRE to FU (INT: +7.7%, *p* < 0.01; CON: +2.1%). Significant time-effects were observed for LE F_max_ (*p* = 0.016; $\eta_{p}^{2}$ = 0.24) and LC P_max_ (*p* = 0.002; $\eta_{p}^{2}$ = 0.35) (see Supplementary Table S7).

**Table 2:** Maximal Strength (F_max_) and Power (P_max_) data for Leg Extension (LE), Leg Curl (LC), and Leg Press (LP) for both groups during PRE, POST, and FU testing, including change (delta).

	Parameter	Group	PRE	POST	% Delta PRE-POST	SMD PRE-POST	FU	% Delta PRE-FU	SMD PRE-FU	ANOVA p ($\eta_{p}^{2}$)	
										Time Effect	Time x Group Interaction
**LE**	F_max_ (N)	INT	2602 (367)	2781 (361)	+6.9**	0.49	2803 (291)	+7.7***	**0**.**61**	**0.016 (0.239)**	**0.029 (0.211)**
		CON	2502 (340)	2514 (229)	+0.5	0.04	2554 (280)	+2.1	0.17		
	P_max_ (W)	INT	1141 (236)	1199 (184)	+5.1	0.28	1225 (223)	+7.3	0.37	0.093 (0.146)	0.947 (0.004)
		CON	1036 (169)	1101 (190)	+6.3	0.36	1123 (189)	+8.4	0.49		
**LC**	F_max_ (N)	INT	1334 (192)	1422 (198)	+6.6	0.45	1455 (233)	+9.1	**0.57**	0.833 (0.012)	0.054 (0.177)
		CON	1348 (176)	1339 (219)	-0.7	-0.05	1364 (164)	+1.2	0.09		
	P_max_ (W)	INT	718 (205)	774 (130)	+7.8	0.32	832 (190)	+15.9	**0.58**	**0.002 (0.350)**	0.371 (0.064)
		CON	647 (97)	712 (114)	+10.1	**0**.**61**	719 (109)	+11.0	**0.69**		
**LP**	F_max_ (N)	INT	3255 (555)	3526 (769)	+8.3	0.40	3744 (829)	+15.0	**0.69**	0.513 (0.043)	0.690 (0.024)
		CON	2905 (566)	3339 (671)	+14.9	**0**.**70**	3517 (752)	+21.1	**0.92**		
	P_max_ (W)	INT	1674 (285)	1710 (324)	+2.2	0.12	1774 (279)	+6.0	0.35	0.229 (0.094)	0.326 (0.072)
		CON	1361 (251)	1498 (289)	+10.1	**0**.**51**	1497 (260)	+10.0	**0.53**		

*Values are presented as mean (±SD). Moderate to large standardized mean differences (SMD) have been highlighted in bold. Significance level was set at ^∗∗^*p* < 0.01 and ^∗∗∗^*p* < 0.001. Eta squared for time effects and interaction are presented in brackets.*

### Jump and Sprint Parameter

Jump and sprint values for both groups are provided in Table 3. All data are adjusted for baseline differences. A significant and large time × group interaction (*p* = 0.007; $\eta_{p}^{2}$ = 0.32) was only observed for 30 mLSp where *post hoc* comparisons indicated a significant decline from PRE to POST and a significant improvement from POST to FU only in the INT group. Significant time-effects were observed during CMJ (*p* = 0.038; $\eta_{p}^{2}$ = 0.20), DJH (*p* = 0.003; $\eta_{p}^{2}$ = 0.33) and 30 mPSp (*p* = 0.049; $\eta_{p}^{2}$ = 0.21) (see Supplementary Table S8).

**Table 3:** Squat Jump (SJ), Counter Movement Jump (CMJ), Drop Jump Height (DJH), Drop Jump Contact Time (DJCT), Standing Long Jump (SLJ), 30 m Linear Sprint (30 mLSp), and 30 m Pendulum Sprint (30 mPSp) for both groups during PRE, POST, and FU testing, including change (delta).

	Parameter	Group	PRE	POST	% Delta PRE-POST	SMD PRE-POST	FU	% Delta PRE-FU	SMD PRE-FU	ANOVA p ($\eta_{p}^{2}$)	
										Time Effect	Time x Group Interaction
**Jumps**	SJ (cm)	INT	36.34 (6.09)	38.28 (5.73)	+5.3	0.33	40.17 (6.19)	+10.5	**0.62**	0.313 (0.075)	0.942 (0.004)
		CON	34.89 (3.02)	36.57 (5.59)	+4.8	0.37	38.48 (4.30)	+10.3	**0.97**		
	CMJ (cm)	INT	42.48 (7.46)	41.93 (6.21)	-1.3	-0.08	43.50 (6.93)	+2.4	0.14	**0.038 (0.195)**	0.664 (0.027)
		CON	39.18 (4.36)	41.06 (4.89)	+4.8	0.41	41.03 (4.92)	+4.7	0.40		
	DJH (cm)	INT	31.60 (4.94)	33.16 (3.74)	+4.9	0.36	33.63 (3.44)	+6.4	0.48	**0.003 (0.328)**	0.971 (0.002
		CON	31.50 (4.10)	32.74 (3.94)	+3.9	0.31	33.40 (3.34)	+6.0	**0.51**		
	DJCT (cm)	INT	178.89 (11.88)	177.22 (13.76)	-0.9	-0.13	177.67 (16.66)	-0.7	-0.08	0.995 (0.000)	0.834 (0.012)
		CON	166.78 (16.97)	161.89 (21.20)	-2.9	-0.26	165.22 (21.91)	-0.9	-0.08		
	SLJ (cm)	INT	225.33 (17.99)	232.22 (15.50)	+3.1	0.41	240.11 (17.97)	+6.6	**0.82**	0.429 (0.055)	0.691 (0.024)
		CON	224.00 (14.82)	235.33 (17.59)	+5.1	**0**.**70**	240.56 (21.02)	+7.4	**0.91**		
**Sprints**	30 mLSp(s)	INT	4.16 (0.10)	4.29 (0.10)	+3.1**	**1**.**30**	4.18 (0.13)	+0.5	0.17	0.119 (0.151)	**0.007 (0.319)**
		CON	4.24 (0.22)	4.23 (0.20)	-0.2	-0.05	4.19 (0.17)	-1.2	-0.25		
	30 mPSp(s)	INT	7.19 (0.19)	7.19 (0.30)	0.0	0.00	7.13 (0.29)	-0.8	-0.25	**0.049 (0.207)**	0.437 (0.062
		CON	7.25 (0.36)	7.11 (0.25)	-1.9	-0.45	7.11 (0.25)	-1.9	-0.45		

Values are presented as mean (±SD). Moderate to large SMD have been highlighted in bold. Significance level was set at ^∗∗^*p* < 0.01. Eta squared for time effects and interaction are presented in brackets.

## Discussion

This study investigated the effects of an 8-week dynamic submaximal superimposed WB-EMS training on strength and power parameters of the leg muscles as well as on jumping and sprinting performance. It was hypothesized that WB-EMS training would provide greater training adaptations than a similar strength training without superimposed WB-EMS. The main findings of this study indicate that (a) superimposed dynamic WB-EMS does not provide greater benefits than dynamic resistance training alone and that (b) transfer effects on jumping and sprinting performance seem to be restricted in both groups.

Previous EMS studies solely compared WB-EMS training to traditional strength training with non-comparable standardizations. Our study used a standardization procedure with similar parameters for both groups regarding exercises, number of repetitions, number of sets, ROM, movement velocity and RPE. The present intervention program further revealed that WB-EMS training over 8-weeks twice a week led to improvements ranging from 8 to 15% in F_max_ and from 5 to 16% in P_max_ on average. The dynamic training program without superimposed WB-EMS led to improvements from 2 to 21% (F_max_) and 8 to 11% (P_max_) on average. The EMS-related improvements in F_max_ are comparable with previous results for local muscles with superimposed EMS obtained from Nobbs and Rhodes (1986) and Portmann and Montpetit (1991) who conducted dynamic training sessions. Studies which conducted isometric electrical stimulations at the maximal individual pain threshold could showed even higher improvements in F_max_ ranging from +33 ± 18% (Selkowitz, 1985; Cabric and Appell, 1987; Miller and Thépaut-Mathieu, 1993; Colson et al., 2000). Smaller improvements in our study might be attributed to the submaximal EMS intensity, which was selected to perform dynamic exercises. A significant time × group interaction effect was merely observed for F_max_ on LE. Despite a lack of significance level of the interaction effect, we at least observed moderate to large effect sizes for F_max_ on LC. However, subsequent pairwise comparison indicated that INT benefited from WB-EMS small to moderate extend (SMD = 0.57). Therefore, the use of superimposed WB-EMS might be a beneficial means to further improve maximal strength in the quadriceps and in tendency in hamstring muscles. Additional motor unit recruitment through EMS with (a) a continuous and exhausting contractile activity in the same pool of motor units during the entire exercise period, (b) a supramaximal temporal recruitment imposed by the high stimulation frequency chosen, and (c) a synchronous recruitment of neighboring muscle fibers might account for these strength gains (Requena Sánchez et al., 2005).

The EMS specific improvements of the hamstring muscles seem to be particularly important and interesting. Hamstring injuries are the most common muscle injuries in team sports (Ekstrand et al., 2011). A meaningful association between the susceptibility of hamstring injuries and a low hamstring/quadriceps ratio has been early proposed (Orchard et al., 1997). The activation of the hamstrings as well as quadriceps muscles were potentially higher with superimposed EMS during all exercises, especially during exercises like split squats or glute-ham rises in which the quadriceps or hamstrings have a primary agonistic or even antagonistic function. Therefore, the simultaneous stimulation of agonistic and antagonistic working muscles through WB-EMS does not seem to be as counterproductive as assumed in order to improve F_max_ and P_max_. The antagonistic working muscles have to adapt to the EMS-induced resistance during eccentric and concentric contractions. This might be especially favorable during the concurrent application of EMS in eccentric muscular actions (Requena Sánchez et al., 2005).

The findings of the present study indicated that the highest improvements in F_max_ and P_max_ occurred 2 weeks after the training intervention. The calculation of SMD revealed larger effect sizes in all strength and power parameters for PRE-FU compared to PRE-POST for the INT group. The delayed adaptations caused by WB-EMS are also described in previous WB-EMS studies from Dörmann (2011) and Wirtz et al. (2016). Consequently, even a submaximal dynamic WB-EMS training seems to need prolonged regeneration periods after training to reach maximal adaptations. These prolonged adaptations might be explained with the accentuated activation of fast motor units at relatively low force levels (Gregory and Bickel, 2005) and the continuous and exhausting contractile activity in the same pool of motor units during the entire exercise period (Requena Sánchez et al., 2005). Based on these results, it can be speculate if a longer regeneration period after training leads to greater adaptations. If so, this would be an indication to apply EMS intermittently to realize similar improvements with a reduced training volume. Maffiuletti et al. (2000) were able to achieve and maintain significant improvements in various strength parameters within 4 weeks after a 4-week intervention with maximal isometric local EMS. Additionally, superimposed WB-EMS offers the opportunity to might shorten the duration of strength training through the intensification of traditional voluntary strength training without increasing the number of training sessions per week (Filipovic et al., 2012). The high attendance rate in the training sessions of the present study indicates that all participants were able to cope with the physical requirements. In particular the WB-EMS training with a submaximal EMS intensity of about 70% of the maximal individual pain threshold seems to be a beneficial compromise to achieve strength and power adaptations as well as to have an appropriate exertional tolerance.

Despite the considerable gains in F_max_ and P_max_, the improvements in the jumping and sprinting tests were restricted. The only significant time × group interaction effect was observed for 30 mLSp with a significant decline in sprinting performance from PRE to POST and a significant improvement from POST to FU for the INT. These results are in consonance with further studies that observed a change in sprint performance after performing dynamic movements with superimposed WB-EMS (Filipovic et al., 2016). However, the sport-specific training orientation of the jumping and sprinting exercises with superimposed WB-EMS did not lead to the suspected results in the majority of the secondary endpoints. Nevertheless, previous studies indicate that dynamic EMS should be combined with additional athletic or plyometric training to better transfer the strength gains into movements like jumping or sprinting (Maffiuletti et al., 2002; Requena Sánchez et al., 2005; Filipovic et al., 2012). Maffiuletti et al. (2002) conducted a combination of separate isometric EMS sessions and separate plyometric jumping sessions during a 4-week training period and improved SJ performance by +21%. Studies that enhance sprint performance also used EMS in combination with separate sprint-specific or plyometric training (Brocherie et al., 2005; Herrero et al., 2006, 2010). In order to improve sport-specific abilities, the simultaneous activation of agonistic and antagonistic working muscles through WB-EMS in combination with jumping or sprinting exercises at the same time does not appear to be the most effective method as shown in the present study. It can be assumed that the recruitment pattern of WB-EMS disturbs the complex coordination of voluntary muscle activation during explosive performed jumps or sprints. The possible advantage of the simultaneous recruitment pattern of WB-EMS for maximal strength improvements with a reduced co-activation of antagonistic muscles consequently seems to be questionable. However, another explanation for the reduced adaptations of jumping and sprinting abilities could be the short regeneration time after the intervention. In our study, all parameters of the INT group increased within 2 weeks after completion of the WB-EMS intervention from POST-FU. It can be assumed, that strength and power improvements due to EMS training cannot be immediately transferred into complex sport-specific movements despite a sport-specific orientation of training exercises. Voluntary recruitment patterns of special movements are necessary and time for a conversion phase of WB-EMS induced improvements take at least 2 weeks. Furthermore, recent studies suggest that the strength gains achieved with WB-EMS may require a longer adaptation period (>7 weeks) or a higher number of EMS sessions per week to be transferred into sport specific movements (Filipovic et al., 2016).

Regarding future research, some limitations of the present study need to be addressed. Due to the relatively complex study protocol including 12 weeks of training/testing and supervised training twice a week for every participant, only a small sample size has been generated. A larger sample size could have increased the study power and might have provided more conclusive results. The second limitation is that the training stimulus of the exercise “Bulgarian Split Squat” was different between INT and CON. Training intensity of both intervention groups was controlled by the Borg RPE-scale and set to >16 (>“hard”). With regard to previous results from Wirtz et al. (2016) which showed that high mechanical loads in combination with WB-EMS do not lead to greater adaptations than a similar training without WB-EMS, training intensity should rather be intensified with the exercise itself or the intensification of the electrical stimulation than with additional weight. Even if this approach was feasible for the INT group, the participants of the CON group needed additional weights for the split squat exercise after 3 weeks of training because of the training progression reaching 16 at the Borg RPE-Scale.

## Conclusion

In conclusion, it seems that dynamic submaximal superimposed WB-EMS training does not provide notable additional improvements in maximal strength and power parameters of the leg muscles of moderately trained, young athletes compared with a similar training intervention without superimposed WB-EMS. Only in the LE F_max_, the INT group could achieve greater improvements than the CON group. Improvements in complex sport-specific movements like jumping or sprinting are restrictive despite a sport-specific orientation of the training exercises.

## Author Contributions

HK, FM, UD, and NW contributed to conception and design of the study. FM, UD, and NW organized and conducted the training intervention and data acquisition. FM and LD performed the statistical analysis. FM wrote the first draft of the manuscript. All authors contributed to manuscript revision, read, and approved the submitted version.

## Conflict of Interest Statement

The authors declare that the research was conducted in the absence of any commercial or financial relationships that could be construed as a potential conflict of interest.
