# Supplementary material for: Effects of an Eight-Week Superimposed Submaximal Dynamic Whole-Body Electromyostimulation Training on Strength and Power Parameters of the Leg Muscles: A Randomized Controlled Intervention Study
Source: Front Physiol. 2018 Dec 5;9:1719. doi: 10.3389/fphys.2018.01719 (PMC6290057; doi:10.3389/fphys.2018.01719)
Supplement: Supplementary file 1 [file Data_Sheet_1.pdf]

Supplementary Material

Effects of an Eight-Week Superimposed Submaximal Dynamic Whole-Body Electromyostimulation Training on Strength and Power Parameters of the Leg Muscles: A Randomized Controlled Intervention Study.

Florian Micke, Heinz Kleinöder, Ulrike Dörmann, Nicolas Wirtz, Lars Donath*

***Correspondence:**

Prof. Dr. Lars Donath, PhD
Department of Intervention Research in Exercise Training
Am Sportpark Müngersdorf 6, 50933 Cologne, Germany
l.donath@dshs-koeln.de
Phone: +49 221 4982 7700

1 Supplementary Tables

Table 1. Training Session 1a – Week 1-3.

**Exercises**		**Reps**	**Sets**	**Rest period [s]**	**ECC:ISO:CON:ISO / movement velocity [s]**
Bulgarian Split Squad	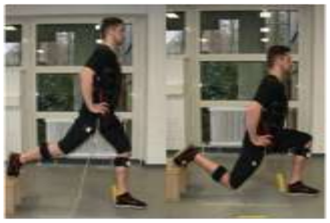	10 e.s.	3	60	2:0:2:1
Russian Leg Curl with support	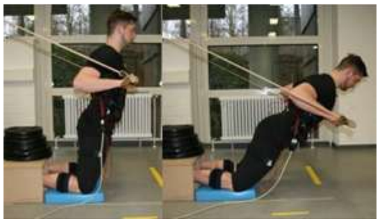	8	3	60	2:0:2:0
Lateral Jumps	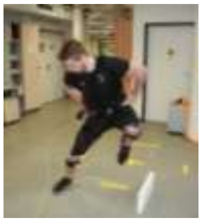	5 e.s.	3	30	explosive
Hurdle Jumps	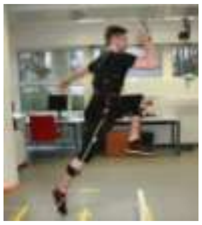	5 e.s.	3	30	explosive
Drop Jumps	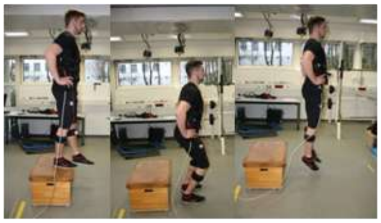	5	3	30	reactive

ECC=eccentric; ISO=isometric; CON=concentric; e.s.=each side

Table 2. Training Session 1b – Week 1-3.

Exercises		Reps	Sets	Rest period [s]	ECC:ISO:CON:ISO / movement velocity [s]
Bulgarian Split Squad	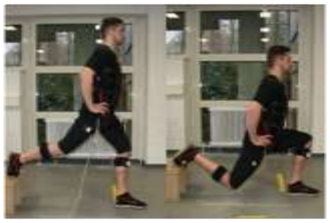	10 e.s.	3	60	2:0:2:1
Russian Leg Curl with support	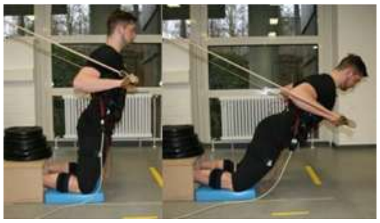	8	3	60	2:0:2:0
Skippings	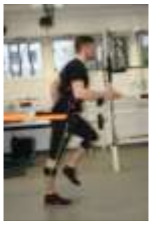	10 s	3	30	max. movement velocity
Hurdle Jumps	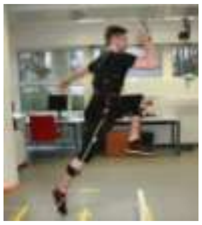	5 e.s.	3	30	explosive
ABC Running Drills - I	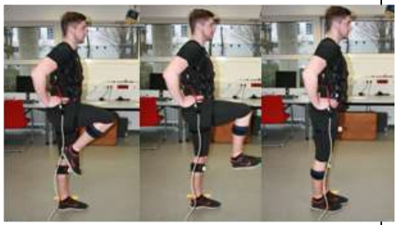	10 e.s.	3	30	max. movement velocity

ECC=eccentric; ISO=isometric; CON=concentric; e.s.=each side

Table 3. Training Session 2a – Week 4-6.

**Exercises**		**Reps**	**Sets**	**Rest period [s]**	**ECC:ISO:CON:ISO / movement velocity [s]**
Bulgarian Split Squad (Slingtrainer)	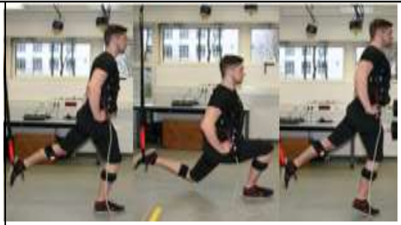	10 e.s.	3	60	2:0:2:1
Leg Curl (Slingtrainer)	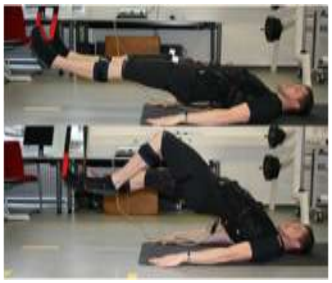	8	3	60	2:0:2:0
Cross Jumps	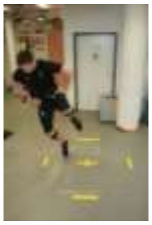	5 e.s.	3	30	explosive
3 Hurdle Jumps	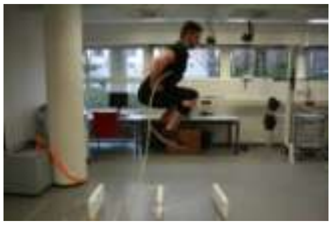	5	3	30	explosive
Standing Long Jump	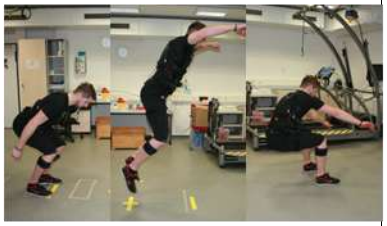	5	3	30	explosive

ECC=eccentric; ISO=isometric; CON=concentric; e.s.=each side

Table 4. Training Session 2b - Week 4-6.

Exercises		Reps	Sets	Rest period [s]	ECC:ISO:CON:ISO / movement velocity [s]
Bulgarian Split Squad (Slingtrainer)	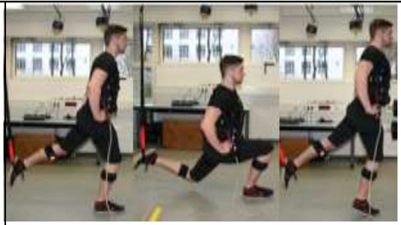	10 e.s.	3	60	2:0:2:1
Leg Curl (Slingtrainer)	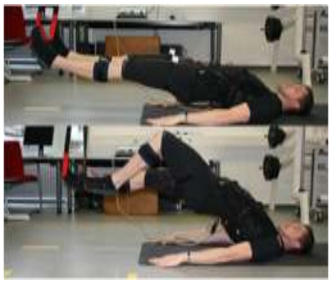	8	3	60	2:0:2:0
Skipping Variations	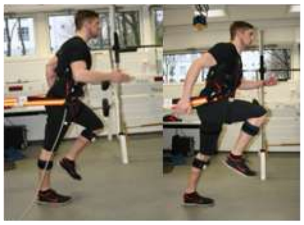	15 s	3	30	
Box Jumps	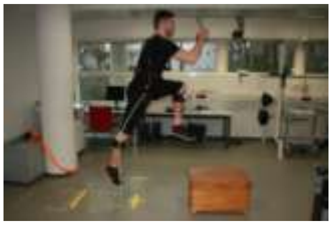	5 e.s.	3	30	explosive
ABC Running Drills - II	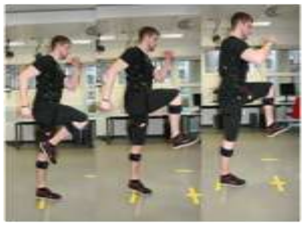	5 e.s.	3	30	max. movement velocity

ECC=eccentric; ISO=isometric; CON=concentric; e.s.=each side

Table 5. Training Session 3a – Week 7-8.

**Exercises**		**Reps**	**Sets**	**Rest period [s]**	**ECC:ISO:CON:ISO / movement velocity [s]**
Bulgarian Split Squad (Slingtrainer)	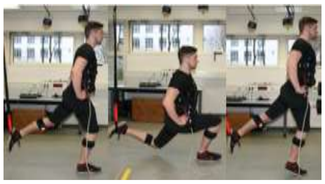	10 e.s.	3	60	3:0:1:1
Leg Curl (Slingtrainer)	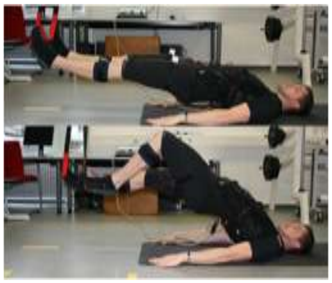	8	3	60	1:0:3:0
Single Leg Box Jumps	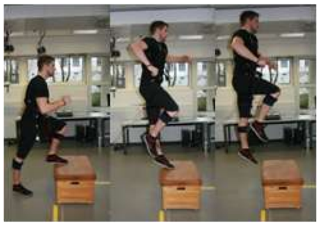	8 e.s.	3	30	explosive
Hurdle Jumps Variation	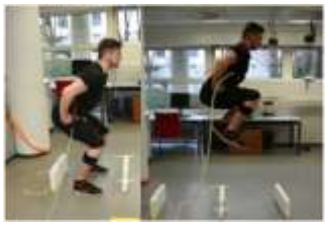	5 e.s.	3	30	explosive/ reactive
Cross Jumps Variation	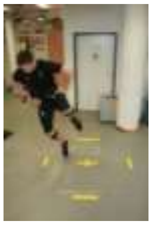	5 e.s.	3	30	explosive/ reactive

ECC=eccentric; ISO=isometric; CON=concentric; e.s.=each side

Table 6. Training Session 3b – Week 7-8.

**Exercises**		**Reps**	**Sets**	**Rest period [s]**	**ECC:ISO:CON:ISO / movement velocity [s]**
Bulgarian Split Squad with Slingtrainer	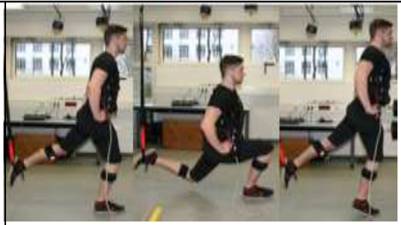	10 e.s.	3	60	3:0:1:1
Russian Leg Curl with Slingtrainer	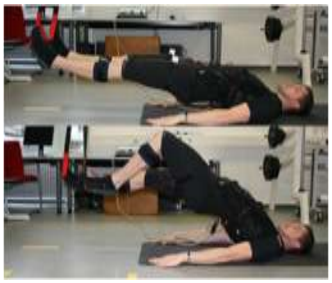	8	3	60	1:0:3:0
Variation Skippings	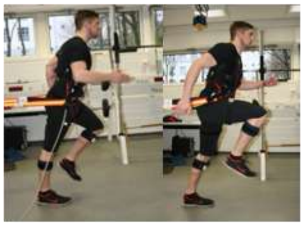	20 s.	3	30	
Single Leg Burpee (forwards)	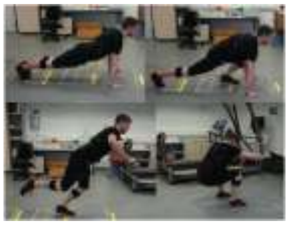	5 e.s.	3	30	explosive
ABC Running Drills -III	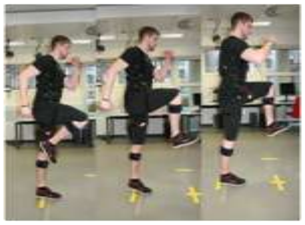	5 e.s.	3	30	

ECC=eccentric; ISO=isometric; CON=concentric; e.s.=each side

Table 7. Maximal Strength (F_{max}) and Power (P_{max}) for the Leg Extension (LE), Leg Curl (LC) and Leg Press (LP) pooled for both groups during PRE, POST, and FU testing, including change (delta).

	Parameter	Group	**PRE**	**POST**	**% Delta** PRE-POST	**FU**	**% Delta** PRE-FU
**LE**	F_{max} (N)	INT & CON	2552 (347)	2647 (324)	+13.7	2678 (305)	+5.0
	P_{max} (W)	INT & CON	1088 (206)	1150 (188)	+5.7	1174 (207)	+7.9
**LC**	F_{max} (N)	INT & CON	1341 (179)	1381 (207)	+3.0	1409 (201)	+5.1
	P_{max} (W)	INT & CON	683 (160)	743 (123)	+8.8	775 (161)	+13.6
**LP**	F_{max} (N)	INT & CON	3080 (573)	3432 (707)	+11.4	3631 (777)	+17.9
	P_{max} (W)	INT & CON	1517 (307)	1604 (317)	+5.7	1635 (298)	+7.8

Values are presented as mean (±SD).

Table 8. Squat Jump (SJ), Counter Movement Jump (CMJ), Drop Jump Height (DJH), Drop Jump Contact Time (DJCT), Standing Long Jump (SLJ), 30 m Linear Sprint (30 mLSp) and 30 m Pendulum Sprint (30 mPSp) pooled for both groups during PRE, POST, and FU testing, including change (delta).

	Parameter	**Group**	**PRE**	**POST**	**% Delta** PRE-POST	**FU**	**% Delta** PRE-FU
**Jumps**	SJ (cm)	INT & CON	35.62 (4.72)	37.42 (5.56)	+5.1	39.32 (5.25)	+10.4
	CMJ (cm)	INT & CON	40.83 (6.16)	41.49 (5.44)	+1.6	42.27 (5.97)	+3.5
	DJH (cm)	INT & CON	31.55 (4.41)	32.95 (3.73)	+4.4	33.52 (3.30)	+6.2
	DJCT (cm)	INT & CON	172.83 (15.52)	169.56 (19.05)	-1.9	171.44 (19.94)	-0.8
	SLJ (cm)	INT & CON	224.67 (16.00)	233.78 (16.16)	+4.1	240.33 (18.97)	+7.0
**Sprints**	30mLSp (s)	INT & CON	4.21 (0.18)	4.26 (0.16)	+1.2	4.19 (0.15)	-0.5
	30mPSp (s)	INT & CON	7.22 (0.29)	7.15 (0.27)	-1.0	7.12 (0.25)	-1.5

Values are presented as mean (±SD).
